# Small RNA functions in carbon metabolism and virulence of enteric pathogens

**DOI:** 10.3389/fcimb.2014.00091

**Published:** 2014-07-15

**Authors:** Kai Papenfort, Jörg Vogel

**Affiliations:** ^1^Department of Molecular Biology, Princeton UniversityPrinceton, NJ, USA; ^2^RNA Biology Group, Institute for Molecular Infection Biology, University of WürzburgWürzburg, Germany

**Keywords:** sRNA, carbon metabolism, Hfq, CsrA, virulence

## Abstract

Enteric pathogens often cycle between virulent and saprophytic lifestyles. To endure these frequent changes in nutrient availability and composition bacteria possess an arsenal of regulatory and metabolic genes allowing rapid adaptation and high flexibility. While numerous proteins have been characterized with regard to metabolic control in pathogenic bacteria, small non-coding RNAs have emerged as additional regulators of metabolism. Recent advances in sequencing technology have vastly increased the number of candidate regulatory RNAs and several of them have been found to act at the interface of bacterial metabolism and virulence factor expression. Importantly, studying these riboregulators has not only provided insight into their metabolic control functions but also revealed new mechanisms of post-transcriptional gene control. This review will focus on the recent advances in this area of host-microbe interaction and discuss how regulatory small RNAs may help coordinate metabolism and virulence of enteric pathogens.

## Introduction

Bacteria colonize almost every niche on earth. Accordingly, they have developed complex regulatory systems to respond to their environment. In particular, the right choice of nutrients is crucial to thrive in conditions of stress or competition. Pathogenic bacteria are no different in this respect. At the very heart of most infections, the host presents an exquisite source of nutrients for the pathogen. However, the immune response of the host can create a hostile environment demanding precise coordination of stress-related and metabolic genes.

Transcription factors have long been known to link metabolic pathways and virulence gene expression. The highly conserved cAMP receptor protein (CRP) transcription factor, for example, coordinates the uptake and utilization of alternative carbon sources in a process termed carbon catabolite repression (CCR) (Gorke and Stulke, 2008). Mutations in CCR components often have drastic consequences for virulence gene expression (Poncet et al., 2009) and loss of CRP activity, either by mutation or low intracellular cAMP levels, strongly reduces the virulence of *Salmonella enterica* (Curtiss and Kelly, 1987; Teplitski et al., 2006), *Vibrio cholerae* (Skorupski and Taylor, 1997), and *Yersinia* species (Petersen and Young, 2002; Kim et al., 2007).

Besides protein-dependent transcriptional control, RNA-controlled mechanisms have turned out to play important roles in regulating virulence genes (Papenfort and Vogel, 2010). Regulatory RNAs operate at all layers of gene expression, ranging from transcription initiation to translation control and protein activity (Waters and Storz, 2009). The majority of the regulatory RNAs characterized to date act by base-pairing with target mRNAs and are commonly referred to as small regulatory RNAs (sRNAs). This group can be further divided into sRNAs encoded on the opposite strand of the regulated RNA (*cis*-encoded) and those that are transcribed distantly from their targets (*trans*-encoded). These sRNAs have been documented to regulate numerous important processes in bacterial pathogens including outer membrane homeostasis (Papenfort et al., 2006, 2010; Song et al., 2008; Corcoran et al., 2012; Fröhlich et al., 2012), quorum sensing (Lenz et al., 2004; Shao et al., 2013), iron homeostasis (Murphy and Payne, 2007), biofilm formation (Monteiro et al., 2012; Zhao et al., 2013), host-cell contact (Heroven et al., 2008; Sterzenbach et al., 2013; Gruber and Sperandio, 2014), and amino-acid metabolism (Sharma et al., 2011).

Other classes of riboregulators are riboswitches (Serganov and Nudler, 2013) or RNA thermometers (Kortmann and Narberhaus, 2012). Both describe RNA elements typically found in the 5′ UTR (untranslated region) of mRNAs regulating gene expression via structural rearrangements of the RNA. Whereas riboswitches respond to varying availability of metabolites or metals in the cell, RNA thermometers function by sensing changes in temperature. Riboswitches may also produce small RNAs (Vogel et al., 2003) and act as *trans*-acting regulators on mRNAs (Loh et al., 2009). For many pathogenic bacteria, host body temperature is a central signal activating virulence gene expression. RNA thermometers have been shown to contribute to this regulation in enteric bacteria such as *Yersinia pseudotuberculosis* and *Listeria monocytogenes* (Johansson et al., 2002; Bohme et al., 2012), as well as the non-enteric human pathogen *Neisseria meningitidis* (Loh et al., 2013).

Due to the relatively small size of their genes or simply because of incomplete genome annotations riboregulators were often overlooked in traditional genetic screens for virulence determinants. In addition, the fact that most regulatory RNAs may act to fine-tune processes and so give milder phenotypes when mutated than regulatory proteins has also disfavored their identification in virulence screens. However, the recent advent of next-generation sequencing (NGS) techniques has begun to remedy some of these limitations: NGS can provide global maps of RNA expression at nucleotide resolution for any bacterial pathogen of interest, and some of the newly identified sRNAs have already been documented to contribute to microbial virulence (Caldelari et al., 2013).

Evidence for regulatory RNAs being important for the control of virulence and metabolism has also come from the loss-of-function phenotypes of two proteins, Hfq (a.k.a. HF-I protein) and CsrA (carbon storage regulator A). The RNA chaperone, Hfq, is required for virulence in diverse bacterial pathogens and *hfq* mutants usually display pleiotropic defects such as reduced growth rates, altered metabolic profiles and changes in virulence gene expression (Chao and Vogel, 2010; Sobrero and Valverde, 2012). At the mechanistic level, Hfq is known to serve as a “molecular matchmaker” by facilitating base-pairing of sRNAs and target mRNAs but it also protect sRNAs from degradation by cellular ribonucleases (Vogel and Luisi, 2011). In the laboratory, Hfq has proven as a useful tool to precipitate bona-fide sRNAs (Chao et al., 2012 and references therein) and therefore frequently served as starting point for the functional characterization of sRNA regulators.

Likewise, the RNA-binding protein CsrA (a.k.a. RsmA in some organisms) is required for virulence of many pathogens (Lucchetti-Miganeh et al., 2008). Originally described as a pleiotropic regulator of glycogen biosynthesis in *Escherichia coli* (Romeo et al., 1993), CsrA homologs have now been annotated in more than 1500 bacterial species (Finn et al., 2014). Binding of CsrA occurs at GGA-rich elements in the mRNA and commonly results in reduced ribosome association and subsequent mRNA decay (Romeo et al., 2013), though CsrA-mediated gene activation has also been reported (Yakhnin et al., 2013). The key regulators of CsrA activity are CsrB-like sRNAs which act as decoys of the protein. These sRNAs, of which many bacteria encode more than one copy, carry multiple high-affinity sites containing the GGA motif and thereby titrate CsrA away from its target mRNAs (Babitzke and Romeo, 2007).

Recent global studies of other gastrointinal pathogens such as *Helicobacter pylori* (Sharma et al., 2010), *Campylobacter jejunii* (Dugar et al., 2013), and *Clostridium difficile* (Soutourina et al., 2013) have suggested a wealth of potential RNA regulators in these organisms, but if and how these are involved in metabolic processes and infection is mostly unclear. Therefore, in this review we concentrated on the functions of established sRNAs in carbon metabolism and virulence of enteric pathogens and, where applicable, outlined the underlying mechanisms of regulation.

## Glucose homeostasis through SgrS

The facultative intracellular pathogen *S. enterica* serovar Typhimurium is probably one of the best understood bacteria when it comes to metabolic profiling during infection (Dandekar et al., 2012). Transcriptome analyses of intracellular *Salmonella* suggested a preference for glucose, glucose-6-phosphate (G-6-P), and gluconate as primary carbon sources during infection (Hautefort et al., 2008); the preference for glucose (though not G-6-P) during intracellular growth was also supported by isotopologue profiling experiments (Gotz et al., 2010). In agreement with these observed preferences, glucose and glycolysis are essential for the virulence of *Salmonella* (Bowden et al., 2009).

**Figure 1:**
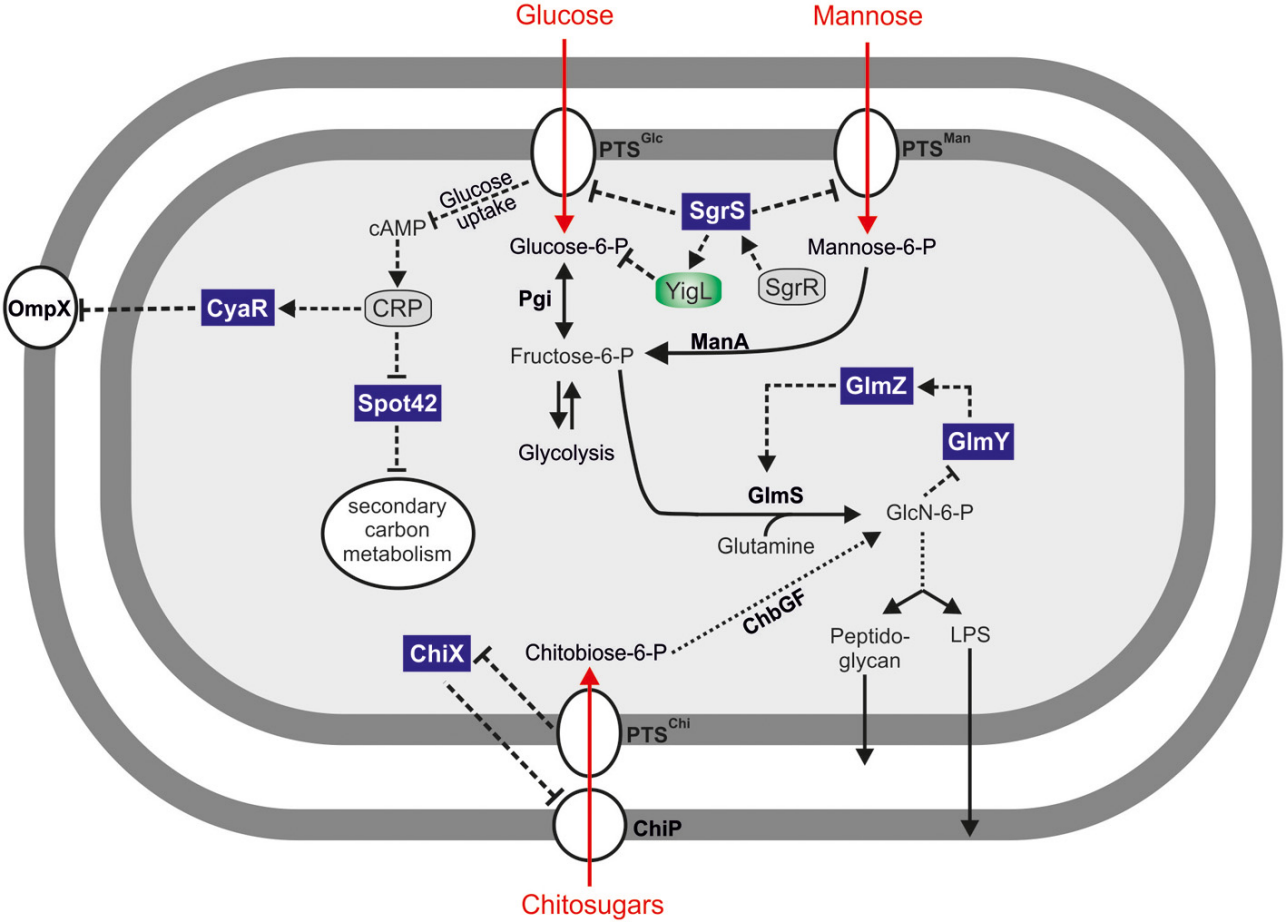
**Hfq-dependent sRNAs regulating carbon metabolism**. Small RNA controlled carbohydrate metabolic pathways for uptake and metabolism of glucose, mannose, and chitosugars. Spot 42 sRNA acts as a global regulator of secondary carbon metabolism. SgrS sRNA controls uptake and secretion of various carbohydrates. The GlmYZ sRNAs control the expression of glucosamine-6-phosphate synthetase (GlmS) in response to its product, GlcN-6-P. Enzymes and transporters are depicted in bold and the transcriptional regulators SgrR and CRP are shown in gray.

Glucose uptake and catabolism are strictly controlled, and *Salmonella* shares many of the underlying regulatory mechanisms with its close relative, *E. coli*. The transport of glucose across the bacterial membrane is achieved by so-called phosphotransferase systems (PTS) (Jahreis et al., 2008). Gram-negative model bacteria encode a plethora of PTS with varying substrate specificities (Deutscher et al., 2006). For glucose, the translocation process generates G-6-P (Figure 1) which, once in the cytosol, can enter several metabolic pathways including glycolysis or the pentose-phosphate pathway.

**Figure 2:**
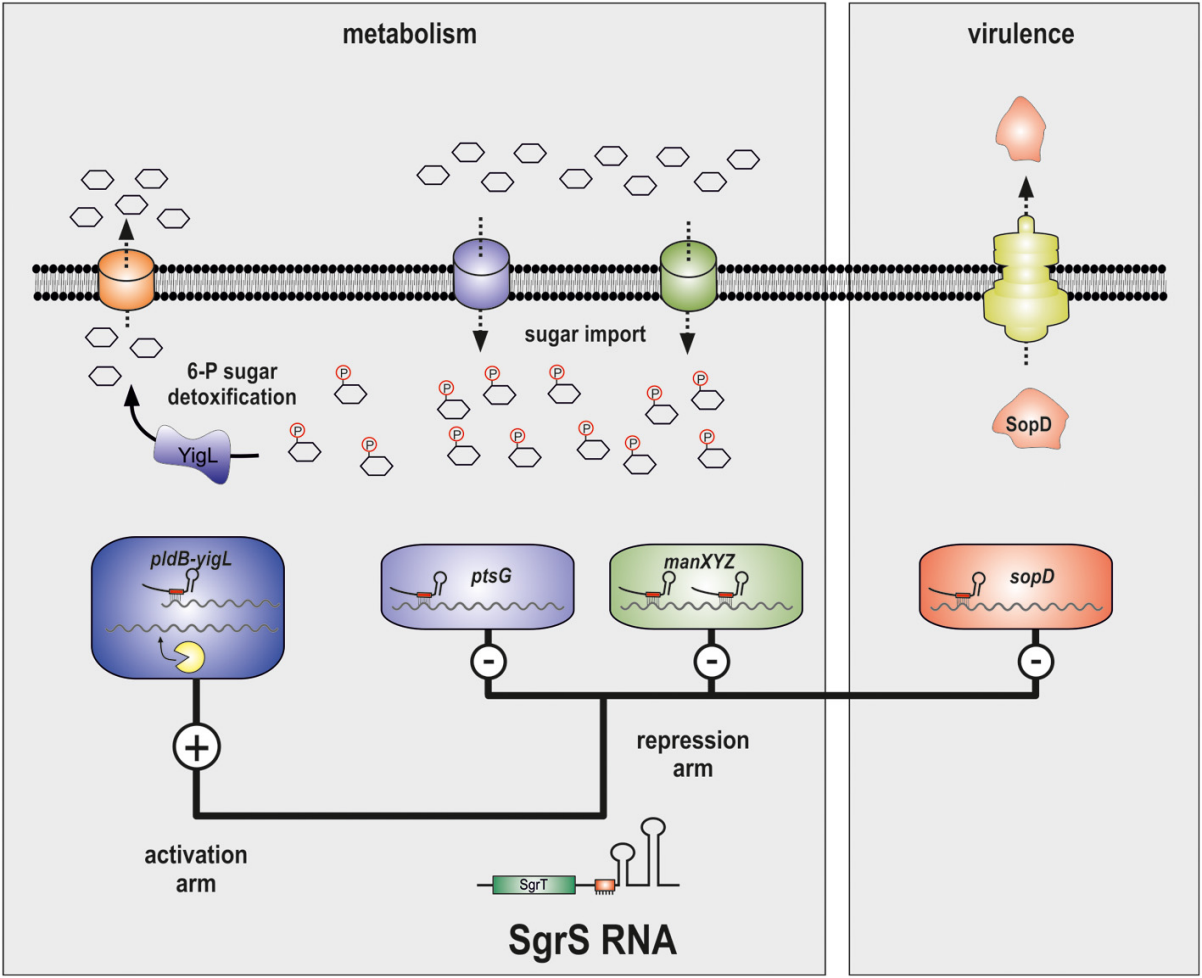
**SgrS controls carbon metabolism and virulence factor production**. The SgrS sRNA regulates the *ptsG* and *manXYZ, yigL* and *sopD* mRNAs via direct base-pairing with the respective transcripts. Activation of *yigL* requires inhibition of endonucleolytic degradation through sequestration of a RNase E cleavage site. The *ptsG* and *manXYZ* mRNAs encode carbohydrate transporters for glucose and mannose, respectively. The *yigL* gene encodes a potent phosphatase which removes phosphate residues from intracellular carbohydrates which allows export. The *sopD* gene is specific to *Salmonella* and its translation results in a secreted virulence factor that enters the mammalian host cell.

Phosphosugars such as G-6-P are a double-edged sword, though. On the one hand, they serve as a primary energy source for generating ATP and NADH via glycolysis. On the other hand, high levels of phosphorylated sugars can impair growth (Irani and Maitra, 1977; Kadner et al., 1992) and may cause DNA damage (Lee and Cerami, 1987). Importantly, many non-metabolizable carbohydrates are invariably imported and phosphorylated by Crr and PtsG, the major proteins for glucose uptake in *E. coli* and *Salmonella*. The accumulation of intracellular G-6-P or other phosphorylated sugars is often referred to as phosphosugar stress and has been observed in many Gram negative bacteria (Bobrovskyy and Vanderpool, 2013). Not surprisingly, intracellular glucose levels are strictly controlled and glucose homeostasis is subject to complex transcriptional and post-transcriptional control. Six transcriptional regulators, including the two alternative sigma-factors σ^S^ and σ^H^, control the *ptsG* gene in *E. coli* (Jahreis et al., 2008). Furthermore, the *ptsG* mRNA is destabilized in response to high intracellular G-6-P levels (Kimata et al., 2001), an effect which could be attributed to the activity of a phosphosugar stress-induced sRNA, SgrS (Vanderpool and Gottesman, 2004). Upon activation by the SgrR transcriptional regulator (Vanderpool and Gottesman, 2004, 2007), SgrS base-pairs with the ribosome binding site (RBS) of the *ptsG* mRNA to inhibit translation initiation. Thereby SgrS reduces *de novo* production of PtsG protein and limits glucose import and intracellular G-6-P levels (Vanderpool and Gottesman, 2004) (Figures 1, 2).

SgrS has many characteristics of an Hfq-dependent sRNA: it co-immunoprecipitates with Hfq (Zhang et al., 2003) and mutation of the *hfq* gene impairs the intracellular stability of SgrS and its ability to repress the *ptsG* mRNA (Kawamoto et al., 2006). Recent work showed that Hfq binds at the Rho-independent transcriptional terminator hairpin at the 3′ end of SgrS (Otaka et al., 2011; Ishikawa et al., 2012). SgrS has also been a model sRNA in establishing general mechanisms of sRNA activity in bacteria. For example, the Aiba group showed that successful repression of *ptsG* by SgrS required a very short seed pairing, involving as few as six essential base-pairs (Kawamoto et al., 2006; Maki et al., 2010); that regulation may occur at the inner membrane (Kawamoto et al., 2005); and crucially involves RNase E (Morita et al., 2005). Interestingly, although SgrS induces *ptsG* mRNA decay (Morita et al., 2005), RNA duplex-formation alone suffices for translational repression (Morita et al., 2006).

Additional mRNA targets of SgrS have been identified (Papenfort et al., 2012). The *manXYZ* transcript encodes a mannose-specific uptake system and is also repressed by SgrS (Figures 1, 2; Rice and Vanderpool, 2011). However, different from the *ptsG* mRNA, the *manXYZ* transcript contains two functional SgrS binding sites (Rice et al., 2012).

In *Salmonella*, SgrS also represses the *sopD* mRNA (Figure 2) (Papenfort et al., 2012) which encodes a *Salmonella*-specific effector protein that is injected into host cells (Brumell et al., 2003). In the host, SopD contributes to phagosome formation (Bakowski et al., 2007) and fluid secretion (Jones et al., 1998). Intriguingly, repression of the *sopD* mRNA requires base-pairing of SgrS to the RBS with the same conserved seed sequence that targets *ptsG* and *manXYZ* (Kawamoto et al., 2006; Papenfort et al., 2012; Rice et al., 2012). Thus, the SgrS seed sequence underlying the regulation of sugar transport mRNAs has been recruited to control the mRNA of a horizontally acquired virulence gene.

The study of this non-sugar stress related *sopD* target has revealed the exceptional fidelity by which SgrS recognizes mRNAs. That is, many *Salmonella* isolates carry a duplication of the *sopD* gene termed *sopD2* (Brumell et al., 2003); the SopD2 protein is also secreted into host cells and is required for virulence in mice (Jiang et al., 2004). However, despite extensive sequence homology between the two virulence factor mRNAs, the *sopD2* mRNA is not regulated by SgrS. Genetic and biochemical analyses of the underlying mechanism revealed that a single-nucleotide variation between the two mRNA sequences is sufficient to prevent SgrS from targeting *sopD*2. This single nucleotide difference renders a stable G-C pair in the productive SgrS-*sopD* interaction into a silent G-U pair which prevents SgrS from regulating *sopD2*. Although the G-U pair is predicted to make only a minor difference in RNA-duplex stability, its crucial location at the proximal end of the RNA seed interaction prevents *sopD*2 from becoming an SgrS target (Papenfort et al., 2012). In other words, a single hydrogen bond (G-C vs. G-U pair) determines which of these two virulence factor mRNAs is regulated by SgrS.

The most recent addition to the list of SgrS target genes is the *yigL* mRNA (Papenfort et al., 2013) (Figure 1). Different from the hitherto known negative regulations, SgrS activates the synthesis of YigL protein (Figure 2). Importantly, the *yigL* gene is expressed as part of a di-cistronic *pldB-yigL* mRNA but the activation by SgrS is restricted to the *yigL* part of the transcript. The underlying activation mechanism involves base-pairing of SgrS to a processed monocistronic *yigL* mRNA species in which SgrS sequesters a RNase E cleavage site. This site-specific inhibition of RNase E-mediated decay increases transcript stability and YigL protein synthesis (Papenfort et al., 2013). This novel mode of post-transcriptional activation complemented previously observed mechanisms of positive regulation (Fröhlich and Vogel, 2009) and was subsequently reencountered in the activation of *cfa* mRNA by RydC sRNA (Fröhlich et al., 2013).

The SgrS-mediated activation of *yigL* plays an important biological role during glucose-phosphate stress. Since it occurs within minutes, it can be considered to be part of an immediate stress response program (Papenfort et al., 2013). The *yigL* gene encodes a potent phosphatase which catalyzes the removal of phosphate residues from intracellular carbohydrates (Kuznetsova et al., 2006; Papenfort et al., 2013). Since the negative charge of the phosphate normally prevents the toxic carbohydrates from crossing the bacterial membrane, the dephosphorylation by YigL enables efficient export and detoxification (Papenfort et al., 2013; Sun and Vanderpool, 2013). Whether the RNA-based activation of *yigL* is important for *Salmonella* infection remains to be seen. However, we note that the *yigL* gene is required for pathogenicity of the insect pathogen *Xenorhabdus nematophila* (Richards et al., 2009).

Another relevant element for instant stress relief is the SgrT peptide. In contrast to most other Hfq-binding sRNAs, SgrS does not strictly act as a non-coding regulator. The proximal part of the molecule encodes the ~40aa SgrT peptide which can inhibit carbohydrate import, likely by blocking the glucose channel (Wadler and Vanderpool, 2007). SgrT is not required for the regulation of target mRNAs (Balasubramanian and Vanderpool, 2013) and not necessarily conserved in *sgrS* homologs of other species (Horler and Vanderpool, 2009).

In order to fully understand the function of SgrS in metabolism and virulence, it will be important to identify the cause of glucose-phosphate stress and the molecule(s) involved in SgrS induction in pathogenic organisms. Suppressor studies in non-pathogenic *E. coli* have suggested a connection of phosphate metabolism and glucose-phosphate stress (Richards and Vanderpool, 2012) and experiments from Aiba and Vanderpool groups indicated that G-6-P itself is not causing toxicity. Rather, the depletion of glycolytic intermediates induces growth arrest (Morita et al., 2003; Richards et al., 2013) but how this ties in with virulence factor control such as the observed repression of SopD synthesis in *Salmonella* remains to be understood.

Along the same line, robust virulence-related phenotypes of *sgrS* mutants are yet to be identified in *Salmonella* (Santiviago et al., 2009; Papenfort et al., 2012). Given the massive competition for glucose by other microbes in the intestine it is likely that SgrS-mediated gene regulation is most relevant when *Salmonella* has entered the host cell. Here, glucose is plentiful and serves as the primary carbon source for intracellular replication (Dandekar et al., 2012). When *Salmonella* disseminates systemically into the liver and spleen it continues to grow within macrophage where glycolysis and glucose metabolism remain highly relevant (Bowden et al., 2009). Therefore, regulation of glycolytic flux and virulence factor production by SgrS could be important under these conditions. In this context it is interesting to note that in *V. cholerae* a related sRNA, TarA, is required for infant mouse colonization by this pathogen. Similar to SgrS, TarA represses the production of PtsG; different from SgrS, though, the expression of TarA is directly controlled by a major virulence transcription factor, ToxT (Richard et al., 2010).

## CRP-controlled sRNAs

Spot 42 (encoded by the *spf* gene) was one of the first bacterial riboregulators identified (Ikemura and Dahlberg, 1973) and it is also one of the most conserved sRNAs (Hansen et al., 2012). Transcription of Spot 42 is repressed by cAMP-bound CRP (Polayes et al., 1988) and the over-expression of this sRNA reduces growth on various carbon sources (Rice and Dahlberg, 1982; Beisel and Storz, 2011). Direct targets of Spot 42 remained elusive until in 2002, when the Valentin-Hansen lab discovered that Spot 42 associated with Hfq (Moller et al., 2002a) and regulated galactose metabolism in *E. coli* (Moller et al., 2002b). Specifically, they showed that Spot 42 targets the distal part of the *galETKM* operon mRNA by base-pairing to the RBS of the *galK* cistron, demonstrating for the first time that sRNAs can post-transcriptionally modulate specific genes within multi-cistronic transcripts. Such discoordinate operon expression, resulting in selective repression or activation of internal cistrons, has recently been described for other sRNAs, too (Balasubramanian and Vanderpool, 2013; Papenfort et al., 2013).

Searches for additional Spot 42 target mRNAs have since revealed a more global role for Spot 42 during glucose catabolism (Beisel and Storz, 2011; Beisel et al., 2012). Nineteen more repressed transcripts were discovered, most of which have documented functions in the transport and metabolism of secondary carbon sources. Intriguingly, many of them are regulated by CRP at the transcriptional level, suggesting that CRP and Spot 42 form a complex feed-forward loop which reinforces CCR. Here, Spot 42 directly inhibits the translation of mRNAs involved in the utilization of secondary carbon sources, the same genes which are also regulated by CRP at the transcriptional level (Beisel and Storz, 2011; Papenfort and Vogel, 2011). Since many target interactions of Spot 42 seem conserved in various enteric pathogens (Wright et al., 2013), Spot 42 may be relevant as carbon source composition change rapidly in the course of an infection process.

CyaR is another CRP-controlled sRNA which binds Hfq and is highly conserved among the enterobacteria (Zhang et al., 2003). In contrast to Spot 42, which is repressed by CRP, CyaR is activated by the CRP-cAMP complex. One conserved target of CyaR is the *ompX* mRNA which encodes a major outer membrane protein of *Salmonella* and *E. coli* (Johansen et al., 2008; Papenfort et al., 2008; De Lay and Gottesman, 2009). Additional targets of CyaR include the transcripts of *yqaE, nadE*, and *luxS* (De Lay and Gottesman, 2009) as well as *ptsI, yobF*, and *sdhA* (Wright et al., 2013), in other words, transcripts of genes that relate directly or indirectly to metabolic functions. For example, the *luxS* gene is required for the production of the common autoinducer AI-2 and repression by CyaR suggests a link between carbon metabolism and population behavior (De Lay and Gottesman, 2009). Recent studies revealed the expression of several CRP-dependent sRNAs (including CyaR) in *Yersinia pestis* -infected lungs, suggesting a potential role for carbon metabolism and sRNAs in pathogenicity (Koo et al., 2011; Yan et al., 2013). Indeed, in *Y. pestis* Crp expression itself depends on the Hfq chaperone which is revelant for the development of pneumonic plague (Lathem et al., 2014).

## Chitin utilization through sRNAs

Chitin is a solid polymer made of N-acetylglucosamine (GlcNAc) and one of the most abundant biomaterials on Earth. Thanks to its inert structure chitin requires specialized enzymes, termed chitinases, to be utilized (Bhattacharya et al., 2007). Interaction with chitin can be important during multi-species biofilm formation with fungal partners and may also affect the virulence of individual bacterial pathogens (Brandl et al., 2011 and references therein). Ecologically, chitinases play an important role in the lifestyle of many marine bacteria, e.g. *V. cholerae* (Meibom et al., 2004) where GlcNAc induces the expression of the competence-regulating TfoR sRNA (Yamamoto et al., 2011). Further, chitinases are also encoded by non-marine enteropathogens such as *Salmonella* (McClelland et al., 2001).

In *E. coli* and *Salmonella* chitin utilization is regulated by a complex mechanism involving the sRNA ChiX (a.k.a RybC, MicM, or SroB) and a decoy mRNA transcript (Mandin and Gottesman, 2009). In the absence of chitosugars, ChiX sRNA continuously binds to and represses the *chiP* mRNA which encodes a chitoporin required for the uptake of chitooligosaccharides (Rasmussen et al., 2009) (Figure 1). Genetic screens for relief of *chiP* repression by ChiX hinted at another layer of post-transcriptional control (Figueroa-Bossi et al., 2009; Overgaard et al., 2009). Here, expression of the *chb* operon (encoding genes for chitosugar utilization) is induced in the presence of chitobiose via the ChbR transcriptional regulator (Plumbridge and Pellegrini, 2004). Through a base-pairing interaction, the *chb* mRNA titrates the ChiX sRNA, inducing a rapid degradation of this repressor. This decoy function of *chb* indirectly increases the synthesis of the ChiP porin, adjusting its levels to the availability of the enzymes for chitosugar processing (Figueroa-Bossi et al., 2009; Overgaard et al., 2009). In addition, when chitosugar concentrations are low ChiX activity is accompanied by transcriptional repression of the *chiP* and *chb* genes by NagC. However, when chitosugars enter the cell repression by NagC is alleviated and *chb* can act as a decoy for ChiX (Plumbridge et al., 2014).

Chitin utilization is also important in the Gram positive bacterium, *L. monocytogenes*. Recent studies suggested that the chitinolytic activity of this pathogen could have important functions during immune evasion; in addition, mutations in the chitinase-encoding gene *chiA* reduced virulence (Chaudhuri et al., 2013b). Interestingly, expression of the *chiA/B* genes is controlled by the master virulence regulator PrfA (Larsen et al., 2010), but the levels of the *chiA* mRNA are additionally controlled by the Hfq-dependent LhrA sRNA (Nielsen et al., 2011). LhrA represses the translation of at least three genes, i.e., *chiA, lmo0302* (hypothetical protein), and *lmo0880* (cell wall associated protein). Expression of LhrA has a negative effect on the chitonolytic activity of *L*. *monocytogenes*, however, it is not yet clear if this function is also relevant for virulence. Note that LhrA was the first example of a sRNA from a Gram positive bacterium that requires Hfq for target regulation (Nielsen et al., 2010).

## The GlmY/Z sRNAs act by sequestration and base-pairing

The two homologous sRNAs, GlmY and GlmZ, are highly conserved among the *enterobacteriae*. Both sRNAs activate production of GlmS (Figure 1), although only GlmZ directly base-pairs with the *glmS* transcript. The *glmS* mRNA accumulates as the distal part of the *glmUS* dicistronic transcript, and is separated from the *glmU* ORF by an RNase E mediated cleavage event (Kalamorz et al., 2007). Following this processing, the *glmS* mRNA remains translationally inactive because of an intrinsic inhibitory structure within its 5′ UTR. Binding of GlmZ to the *glmS* 5′ UTR resolves this structure, which releases the RBS of this transcripts and increases synthesis of the GlmS protein (Reichenbach et al., 2008; Urban and Vogel, 2008).

The enzymatic product of GlmS is glucosamine-6-phosphate (GlcN6P), a central aminosugar required for cell wall biosynthesis. Low levels of GlcN6P induce the expression of GlmY which indirectly activates GlmS production through GlmZ and the accessory protein, RapZ (a.k.a. YhbJ). Due to its structural similarity with GlmZ, GlmY can function through molecular mimicry to interfere with GlmZ degradation by RNase E and RapZ. The latter protein is a specialized adapter that targets GlmZ for RNase E-mediated decay. Recognition by RapZ is guided by a RNA element shared between GlmZ and GlmY and high levels of GlmY titrate the RapZ protein from GlmZ, thus stabilizing the GlmZ sRNA. GlmY itself does not bind Hfq, suggesting that it acts as a specific decoy for GlmZ rather than regulating mRNAs expression on its own (Gopel et al., 2013). Taken together, these two well-conserved sRNAs act hierarchically in a complex regulatory cascade to adjust the translation of the *glmS* mRNA to physiological needs.

In *Salmonella* and other enterobacteria, transcription of GlmY and GlmZ is regulated by two overlapping promoters controlled by either σ^70^ or σ^54^ although this may vary between species (Urban et al., 2007; Reichenbach et al., 2009; Gopel et al., 2011). GlmY expression also requires binding of the global transcriptional regulator, IHF (Gopel et al., 2011). In addition, the expression of the *glmY/Z* genes by the σ^54^ version of RNA polymerase requires the QseF and QseE proteins (a.k.a. GlrR/GlrK) (Reichenbach et al., 2009; Gopel et al., 2011). Intriguingly, QseF and QseE constitute a two-component system that is important for the virulence of *Y. pseudotuberculosis* (Flamez et al., 2008) and enterohemorrhagic *E. coli* (EHEC) (Reading et al., 2007) indicating that GlmY/Z might have a function in virulence.

Indeed, the Sperandio group recently reported a crucial role of the GlmY/Z sRNAs for the pathogenicity of EHEC, observing that mutations of either *glmY* or *glmZ* increased pedestal formation on host cells by this organisms (Gruber and Sperandio, 2014). Surprisingly, GlmY/Z did not seem to control *glmS* expression in EHEC. Instead, both sRNAs regulated transcripts from the LEE4 and LEE5 pathogenicity islands as well as the mRNA of the secreted effector protein EspFu. This regulation is reminiscent of the above described SgrS-*sopD* example (Papenfort et al., 2012) in that conserved “core” sRNAs are recruited to regulate the mRNAs of horizontally acquired virulence factors through Hfq and base-pairing.

## Global functions for the RNA-binding protein, CsrA

CsrA-like proteins are conserved in most enteric pathogens and deletion of the *csrA* gene often impairs virulence (Lucchetti-Miganeh et al., 2008; Seyll and Van Melderen, 2013). Given the multi-faceted phenotypes of many *csrA* mutant strains, one may argue that reduced pathogenicity primarily resulted from decreased overall fitness rather than the specific virulence functions. Indeed, a *Salmonella csrA* mutant displayed multiple defects in metabolic regulation and virulence factor expression (Altier et al., 2000; Lawhon et al., 2003) and comparable phenotypes were observed in uropathogenic *E. coli* (Mitra et al., 2013). However, CsrA also regulates *Salmonella* pathogenicity more directly. For example, CsrA binds to the 5′ UTR of the mRNA of HilD repressing the synthesis of this master transcriptional regulator of virulence (Martinez et al., 2011). Similarly, CsrA affects biofilm formation through interaction with the mRNA of an phosphodiesterase gene (STM3611) regulating intracellular c-di-GMP levels (Jonas et al., 2010). CsrA was also found to coordinate the expression of two mutually exclusive fimbrial operons in *Salmonella* by a putative novel mechanism of mRNA cross-regulation (Sterzenbach et al., 2013).

The global activity of CsrA in *E. coli* and *Salmonella* is counteracted by the CsrB/C sRNAs whose transcription is under control of the BarA/UvrY TCS (Gudapaty et al., 2001; Suzuki et al., 2002). Transcriptional control of the CsrA antagonists by the BarA/UvrY TCS seems to be a conserved principle in many bacteria (Seyll and Van Melderen, 2013). While some bacteria encode only one CsrB-like RNA, *V. cholerae* species encode three different CsrA antagonists: CsrB, CsrC, and CsrD (Lenz et al., 2005). Here, expression of the Csr-sRNAs affects virulence via regulation of the quorum sensing pathway (Jang et al., 2010). In addition, expression of CsrB-like sRNAs can also be controlled post-transcriptionally. The CsrD RNA-binding protein of *E. coli* (not to be confused with the CsrD sRNA from *V. cholerae*) can bind the CsrB/C sRNAs and target them for degradation by RNase E (Suzuki et al., 2006). CsrA also reduces the expression of CsrD (Jonas et al., 2008) generating a negative feedback loop for robust signaling under conditions of stress (Adamson and Lim, 2013).

The Csr system and its relevance for virulence and metabolism have been studied in greater detail for the human enteropathogen *Y. pseudotuberculosis* where a mutation of the *csrA* gene resulted in complex phenotypic alterations (Heroven et al., 2008). Transcriptomic studies revealed deregulation of ~500 ORFs in the *csrA* mutants, ~20% of which are metabolic genes (Heroven et al., 2012a). The *Y. pseudotuberculosis* genome encodes two CsrB-like sRNAs (CsrB and CsrC) and their expression is crucial during the initial phase of infection because sequestration of CsrA is needed to allow the production of the host cell adhesion factor, InvA (Heroven et al., 2008). Induction of InvA involves a complex regulon including the transcriptional factor RovA (Heroven and Dersch, 2006). Regulation via the Csr-system is further controlled via CCR. The CRP protein represses the response regulator UvrY which is required for CsrB activation. A *crp* mutant has increased levels of the CsrB sRNA which promotes CsrC and RovA repression. Not surprisingly, a *Y. pseudotuberculosis* mutant lacking the *crp* gene is strongly impaired in virulence (Heroven et al., 2012b).

**Figure 3:**
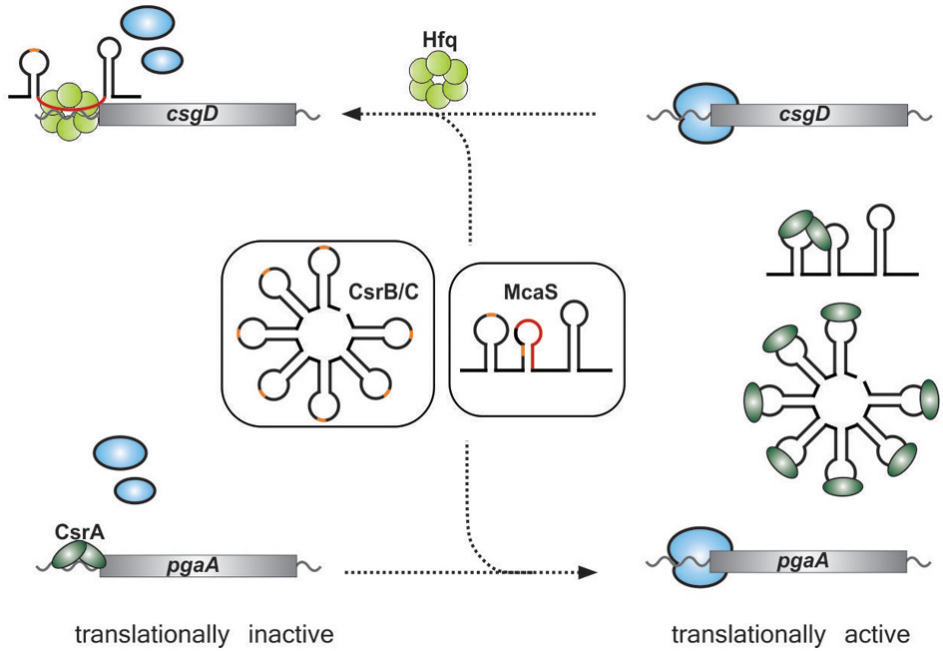
**Overlap of the CsrA and Hfq regulons through McaS**. Both McaS and the CsrB-like sRNAs bind to the CsrA protein via GGA-rich motifs (orange). Binding of CsrA results in titration of CsrA from its target mRNAs which usually activates their translation (lower panel). In addition, McaS can also directly bind and regulate target mRNAs (e.g. *csgD*) through Hfq-mediated base-pairing (indicated in red).

A new type of CsrA antagonist has recently been reported in *E. coli*. It was observed that the Hfq-binding sRNA, McaS, which regulates the *fhlD* and *csgD* mRNAs (encoding regulators of motility and biofilm formation, respectively) by base pairing interactions, impacted expression of the *pgaA* gene by a supposedly indirect mechanism (Jorgensen et al., 2012; Thomason et al., 2012) (Figure 3). The PgaA protein is crucial for the production of PGA (poly-β-1,6-N-acetyl-glucosamine), an important factor for biofilm adhesion (Itoh et al., 2008). Expression of *pgaA* had been known to be subject to control by CsrA (Wang et al., 2005), which suggested a link between McaS and CsrA. Indeed, the McaS sRNA was found to bind the CsrA protein via two exposed GGA motifs and thereby indirectly regulate the expression of several CsrA-target genes, including *pgaA* (Jorgensen et al., 2013) (Figure 3). In summary, McaS is the first sRNA regulating target gene expression via both Hfq and CsrA. Future studies may reveal additional sRNAs that serve in both of these global post-transcriptional networks.

## Future directions

The above examples of sRNA-mediated gene regulation in enteric pathogens serve to illustrate the growing number of potential post-transcriptional links between metabolic and virulence functions in these organisms. To date, many of these links remain inferences from functional studies of sRNA-mRNA interactions, and how these contribute to nutritional adjustment and control of virulence factor expression requires more detailed studies. However, it is important to note that global studies of the RNA targets of Hfq and CsrA, two proteins that each may control up to 20% of all mRNAs in enteric model organisms (Chao and Vogel, 2010; Romeo et al., 2013), revealed a high number of mRNAs from metabolic and virulence pathways, suggesting that many more sRNAs could be involved in these pathways. In addition, the growing depths of NGS will soon allow us to extensively profile bacterial RNA expression in complex tissue and inside host cells, even simultaneously with gene expression of the eukaryotic host to inform details of the pathogen's metabolic environment (Westermann et al., 2012).

There are more potential links between virulence and metabolism in the available sRNA data whose physiological importance needs to be explored. For example, the recent profiling of Hfq-bound *Salmonella* transcripts revealed the DapZ sRNA, which is encoded in the 3′ UTR of the well-conserved metabolic *dapB* gene. In *Salmonella*, the horizontally acquired virulence regulator HilD has been recruited to transcriptionally activate the DapZ sRNA which then acts to repress the synthesis of oligopeptide uptake proteins (Chao et al., 2012). Under regular growth conditions oligopeptide uptake is controlled by the conserved GcvB sRNA (Sharma et al., 2011) and regulation of DapZ by HilD enables the cell to exert a similar function under virulence-related conditions. However, why DapZ is linked to *dapB* and how the metabolic function of the DapB protein, an enzyme that produces the lysine precursor diaminopimelate, may be interwoven with a DapZ-mediated repression of amino acid uptake, is far from obvious. Of note, regulation of oligopeptide uptake through sRNAs has been observed in non-enteric bacteria, too. The RsaE sRNA from *Staphyloccus aureus*, which is also conserved in other Gram positives, directly controls the mRNA encoding the OppB protein (Geissmann et al., 2009) and several other transcripts of metabolic genes (Bohn et al., 2010).

The most recent count for sRNA regulators in *Salmonella* revealed ~280 sRNAs, many of which are Hfq-dependent and expressed under stress or virulence mimicking conditions (Kroger et al., 2012, 2013). How many of these sRNAs are also relevant for virulence is still an open question but novel approaches such as Tn-Seq (combining transposon mutagenesis and HTPS) could be powerful tools to evaluate the roles of sRNAs during infection (Van Opijnen and Camilli, 2013). The same technology can also be used to identify metabolic genes required for infection. Indeed, two recent studies using Tn-Seq in *Salmonella* or *V. cholerae* identified several genes involved in carbon metabolism to be required for full pathogenicity (Chaudhuri et al., 2013a; Fu et al., 2013).

Probably one of the most exciting areas of host-microbe interaction today is how pathogens deal with the commensal microbiota of the host. It is now understood that the carbohydrate metabolism of the microbiota significantly impacts on the virulence gene expression of enteric pathogens and that carbohydrates can function as signaling molecules in the intestine (Pacheco et al., 2012). In contrast, close to nothing is known about how sRNAs shape the interaction of pathogens with commensals and we are yet to see if such sRNAs would also impact virulence. Again, NGS-based metatranscriptomics of multi-species intestinal communities could provide a valuable starting point to address the relevance of regulatory RNAs and metabolic genes in the context of the host microbiota (Xiong et al., 2012). These new exciting venues at the interface of microbiology and host-microbe interaction might become relevant for the design of alternative anti-microbial compounds which consider both, the pathogen and the host microbiota.

### Conflict of interest statement

The Guest Associate Editor Thomas Dandekar declares that, despite being affiliated to the same institution as author Jörg Vogel, the review process was handled objectively and no conflict of interest exists. The authors declare that the research was conducted in the absence of any commercial or financial relationships that could be construed as a potential conflict of interest.
